# Bioavailability of Bergamot (*Citrus bergamia*) Flavanones and Biological Activity of Their Circulating Metabolites in Human Pro-Angiogenic Cells

**DOI:** 10.3390/nu9121328

**Published:** 2017-12-06

**Authors:** Valentina Spigoni, Pedro Mena, Federica Fantuzzi, Michele Tassotti, Furio Brighenti, Riccardo C. Bonadonna, Daniele Del Rio, Alessandra Dei Cas

**Affiliations:** 1 Department of Medicine and Surgery, University of Parma, 43126 Parma, Italy; valentina.spigoni@unipr.it (V.S.); federica.fantuzzi1@gmail.com (F.F.); riccardo.bonadonna@unipr.it (R.C.B.); 2 The Laboratory of Phytochemicals in Physiology, Department of Food & Drugs, University of Parma, 43125 Parma, Italy; pedromiguel.menaparreno@unipr.it (P.M.); michele.tassotti@studenti.unipr.it (M.T.); furio.brighenti@unipr.it (F.B.); 3 Division of Endocrinology and Metabolic Diseases, Azienda Ospedaliero-Universitaria of Parma, 43126 Parma, Italy

**Keywords:** bergamot, citrus fruits, phenolic compounds, hesperetin, naringenin, conjugated phase II metabolites, myeloid angiogenic cells, inflammation, lipotoxicity, endothelial dysfunction

## Abstract

Myeloid angiogenic cells (MACs) play a key role in endothelial repairing processes and functionality but their activity may be impaired by the lipotoxic effects of some molecules like stearic acid (SA). Among the dietary components potentially able to modulate endothelial function in vivo, (poly)phenolic compounds represent serious candidates. Here, we apply a comprehensive multidisciplinary approach to shed light on the prospects of Bergamot (*Citrus bergamia*), a citrus fruit rich in flavanones and other phenolic compounds, in the framework of lipotoxicity-induced MACs impairment. The flavanone profile of bergamot juice was characterized and 16 compounds were identified, with a new 3-hydroxy-3-methylglutaryl (HMG) flavanone, isosakuranetin-7-*O*-neohesperidoside-6″-*O*-HMG, described for the first time. Then, a pilot bioavailability study was conducted in healthy volunteers to assess the circulating flavanone metabolites in plasma and urine after consumption of bergamot juice. Up to 12 flavanone phase II conjugates (sulfates and glucuronides of hesperetin, naringenin and eriodyctiol) were detected and quantified. Finally, the effect of some of the metabolites identified in vivo, namely hesperetin-7-*O*-glucuronide, hesperetin-3′-*O*-glucuronide, naringenin-7-*O*-glucuronide and naringenin-4′-*O*-glucuronide, was tested, at physiological concentrations, on gene expression of inflammatory markers and apoptosis in MACs exposed to SA. Under these conditions, naringenin-4′-*O*-glucuronide and hesperetin-7-*O*-glucuronide were able to modulate inflammation, while no flavanone glucuronide was effective in curbing stearate-induced lipoapoptosis. These results demonstrate that some flavanone metabolites, derived from the in vivo transformation of bergamot juice phenolics in humans, may mitigate stearate-induced inflammation in MACs.

## 1. Introduction

Endothelial health relies on a dynamic equilibrium between injury induced by a relevant number of cues and *noxae* and its self-repair capacity [1]. Myeloid angiogenic cells (MACs), bone marrow-derived cells also referred to as early endothelial progenitor cells (EPCs), are key mediators of endothelial renewal, acting mainly by secreting pro-angiogenetic growth factors [2]. The number and function of MACs are unfavorably influenced by the presence of metabolic stressors, such as insulin-resistance (IR) and cardiovascular (CV) risk factors and diseases, leading to endothelial dysfunction [3,4].

Lipotoxicity, an hallmark of insulin-resistance (IR) states, is a well-known mechanism underlying the association among IR, endothelial dysfunction and increased CV risk [5,6]. Lipotoxicity is characterized by chronically elevated free fatty acid (FFA) levels, mainly saturated free fatty acids (SFAs), palmitic (16:0) and stearic acid (SA, 18:0), which may damage vessel wall by enhancing inflammation/oxidative stress [7,8] and endothelial cell apoptosis [9,10]. We recently demonstrated [11] that SA at physiological concentrations increased inflammation and apoptosis in MACs, suggesting that lipotoxicity may directly and negatively also affect endogenous endothelial repairing processes. This experimental model might be particularly suitable to assess the effects of molecules/drugs in reducing IR-associated lipotoxicity.

Dietary (poly)phenols are known to ameliorate IR [12,13] and counteract vascular cell injury, pointing to their defensive role in preventing endothelial dysfunction and ultimately CV disease development and progression [14,15,16,17].

Citrus plants are rich sources of flavanones [18], a class of flavonoids that may display anti-inflammatory/-oxidant properties and have been reported to play a protective role in CV diseases [19,20]. Specifically, *Citrus bergamia* Risso et Poiteau, an endemic plant of the Calabria region (Italy) cultivated along the southern coast, commonly named “bergamot” and belonging to the Rutaceae family, is particularly rich in glycosides of hesperetin, naringenin and eriodictyol [18]. Bergamot juice (BJ) has been considered for long time merely a byproduct of essential oil extraction, which is widely used in the cosmetic and food industries. Nevertheless, over the last decade, new evidence emerged on the possible favorable anti-inflammatory [21,22,23], antiproliferative [24], neuroprotective [22] and hypolipidemic [25] properties of BJ derivatives. However, despite this body of evidence, there is a lack of information on the specific phenolic metabolites that might be responsible of the biological activity of bergamot.

To the best of our knowledge, no studies to date have assessed (a) the bioavailability of BJ flavanones and (b) whether their beneficial effects on the endothelial/CV system may be mediated, at least in part, by an improvement in vascular regenerative capacity. Notably, the majority of in vitro studies addressing the effects of bergamot juice may be fraught with methodological issues by testing the juice as such [22,23,24], instead of the circulating derivatives resulting of phase II metabolic transformations. Therefore, the objectives of the present study were: (a) to assess the bioavailability of *Citrus bergamia*-derived phenolic metabolites in healthy humans following BJ intake; and (b) to evaluate the potential role of the identified metabolites in curbing lipoapoptosis and inflammation in MACs obtained from healthy donors, following SA exposure in vitro.

## 2. Materials and Methods

### 2.1. Chemicals

Hesperetin-7-*O*-rutinoside (hesperidin), naringenin-7-*O*-rutinoside (narirutin) and isosakuranetin-7-*O*-rutinoside (didymin) were purchased from Extrasynthese (Genay CEDEX, France). Naringenin-7-*O*-glucuronide and naringenin-4′-*O*-glucuronide were purchased from Bertin Pharma (Montigny le Bretonneux, France), while hesperetin-7-*O*-glucuronide and hesperetin-3′-*O*-glucuronide from Santa Cruz (Santa Cruz, CA, USA). Quercetin-3′-*O*-sulfate was kindly provided by Alan Crozier (University of California, Davis, CA, USA). HPLC grade solvents and stearic acid were obtained from Sigma-Aldrich (St. Louis, MO, USA).

### 2.2. Ethics Statement

The in vitro study was approved by the local Ethics Committee, with protocol n. 42170. The in vivo bioavailability study was ethically approved by the Institutional Review Board (IRB) of the University of Parma with protocol n. 0003/2016 and was conducted in accordance with the Declaration of Helsinki. Relevant principles of Good Clinical Practice were followed throughout the study. All subjects gave written informed consent for the bioavailability study. Conversely, no informed consent was required for the cell culture study, as blood donor material was fully anonymized.

### 2.3. Bioavailability Study

Three healthy adults (2 females and 1 male, mean age 38 ± 3 years) were recruited for the bioavailability study with BJ. Exclusion criteria were age <18 or >50 years, body mass index <18 or >25 kg/m^2^, pregnancy or lactation, celiac or other metabolic diseases, past or current CV diseases, any chronic medication and/or hormone replacement therapy, cigarette smoking, alcohol intake > 80 g/day and use of dietary supplements. Subjects were asked to avoid the consumption of flavonoid-rich foods (e.g., berries, citrus fruits, dark chocolate, green tea and red wine) for the 2 days before entering the study.

The day of the study, participants were invited to drink 400 mL of BJ (Bergasterol^®^) after an overnight fasting. Venous plasma samples were obtained at baseline (before juice consumption) and at 1 h and 4 h after juice intake to assess the circulating phenolic derivatives coming from BJ flavanones. Urine samples were also collected at baseline and at different collection times (0–2 h and 2–6 h). During this period of time, subjects avoided the consumption of flavonoid-rich foods. Blood samples were centrifuged and plasma was collected, aliquoted and stored at −80 °C until further processing. Urine samples were also aliquoted and stored at −80 °C until further processing.

### 2.4. Extraction and UHPLC-MS Analysis of Phenolic Compounds from Bergamot Juice

The phenolic compounds in BJ were extracted according to previous reports, with some modifications [26,27]. Briefly, 50 mg of freeze-dried BJ were mixed with 1 mL of 80% aqueous methanol acidified with formic acid (1%). The mixture was vortexed and sonicated for 30 min. The mixture was centrifuged at 10,480× *g* for 10 min at room temperature and the supernatant was collected. The pellet was then extracted with 0.5 mL of dimethyl sulfoxide and followed the procedures described above, after which it was centrifuged. The supernatant and the pellet extracted were filtered through a 0.22 μm nylon membrane before UHPLC-MS analysis. Each sample was extracted in triplicate.

Samples from both the supernatant and pellet were analyzed using an Accela UHPLC 1250 equipped with a linear ion trap-mass spectrometer (LTQ XL, Thermo Fisher Scientific Inc., San Jose, CA, USA) fitted with a heated-ESI probe (H-ESI-II; Thermo Fisher Scientific Inc.). Separations were performed using a XSELECTED HSS T3 (50 × 2.1 mm), 2.5 µm particle size (Waters, Milford, MA, USA). The volume injected was 5 µL and the column oven was set to 30 °C. Elution was performed at a flow rate of 0.2 mL/min. The gradient started with 95% of 0.1% aqueous formic acid, isocratic conditions maintained for 3 min and then a 9-min linear gradient from 5 to 40% acetonitrile with 0.1% formic acid was applied. From 12 to 13 min the acidified acetonitrile was increased to 80%, followed by 3 min of 80% acetonitrile and 5 min at the start conditions to re-equilibrate the column.

The mass spectrometer was operated in negative mode. Capillary temperature was equal to 275 °C and the source heater temperature was set to 200 °C. The sheath gas flow was 40 units, while the auxiliary gas was set to 5 units. The source voltage was 4 kV. The capillary voltage and tube lens were −42 and −118 V, respectively. Analyses were carried out using full scan mode, data-dependent MS^3^ scanning from *m*/*z* 100 to 1500, with collision induced dissociation (CID) equal to 30 (arbitrary units). Pure helium gas was used for CID. Specific MS^2^ and MS^3^ analyses were carried out to unambiguously identify the flavanones presented in BJ supernatant and pellet. Identification was also performed by comparison with standards, when available and literature. Quantification was performed with calibration curves of pure commercial standards, when available, in selected ion monitoring (SIM) mode by selecting the relative base peak at the corresponding mass to charge ratio (*m*/*z*). Naringenin and eriodyctiol derivatives were quantified as narirutin equivalents, hesperetin derivatives as hesperidin equivalents and isosakuranetin derivatives as didymin equivalents. Data processing was performed using Xcalibur software (Thermo Scientific Inc., San Jose, CA, USA).

### 2.5. Extraction and UHPLC-MS Analysis of Phenolic Compounds in Urine and Plasma Samples

Urine samples were diluted 1:1 (*v*/*v*) adding acidified water (0.1% formic acid *v*/*v*), centrifuged at 14,000 rpm for 10 min and filtered through a 0.45 μm nylon filter before UHPLC-MS analysis. Plasma samples were extracted following the method reported by Ludwig et al. [28]. Briefly, 400 μL of plasma were added to 1 mL of 2% formic acid in acetonitrile. Samples were vortexed for 1 min, centrifuged at 14,000 rpm for 10 min and supernatants were then dried under a rotary-vacuum evaporation (Thermo Fisher Scientific Inc.). The pellet was resuspended with 100 μL of methanol:water:formic acid (50:50:0.1, *v*/*v*/*v*) and centrifuged at 14,000 rpm for 5 min prior to UHPLC-MS analysis.

Processed biological fluids were analyzed using the Accela UHPLC 1250 with a LTQ XL linear ion trap-mass spectrometer fitted with a heated-ESI probe (Thermo Scientific Inc.). Separations were carried out by means of an Acquity UPLC HSS T3 column (100 × 2.1 mm, 1.8 μm particle size, Waters, Milford, MA, USA). For UHPLC, mobile phase A was acetonitrile containing 0.1% formic acid and mobile phase B was 0.1% formic acid in water. The gradient started with 15% A, isocratic conditions were maintained for 0.5 min and reached 60% A after 6.5 min. From 7 to 8 min, the acidified acetonitrile increased to 80% and was kept at 80% for 2 min. The starting gradient was then immediately reestablished and maintained for 4 min to re-equilibrate the column. The flow rate was 0.4 mL/min, the injection volume was 5 μL and the column temperature was set at 40 °C.

The MS was operated in negative ionization mode with a capillary temperature of 275 °C, with the source temperature of 50 °C. The sheath gas flow was 50 units and the auxiliary gas set to 3 units. The source voltage was 3.7 kV. The capillary and tube lens voltage were −33 and −88 V, respectively. Helium gas was used for CID. A preliminary analysis was performed in full scan, data-dependent MS^3^ mode, scanning from a mass to charge (*m*/*z*) of 100 to 1000, with CID of 35 (arbitrary units), to perform an assessment of the main flavanone phase II metabolites present in the samples. On the basis of the obtained information, a second preliminary analysis was carried out in full MS^2^ and MS^3^ mode to confirm the identification of the detected metabolites. Last, quantification of all flavanone metabolites was achieved using the single reaction monitoring (SRM) mode. Data processing was performed using Xcalibur software (Thermo Scientific). Quantification was performed with calibration curves of standards, when available. Eriodyctiol glucuronides were quantified as naringenin-7-*O*-glucuronide equivalents, while all sulfate conjugates as quercetin-3′-*O*-sulfate equivalents.

### 2.6. Cell Culture and Conditions

MACs were isolated and cultured as previously described [29,30,31]. Briefly, peripheral blood mononuclear cells were isolated by Lymphoprep (Euroclone, Milan, Italy) density gradient centrifugation from healthy volunteers’ buffy-coats. A total of 10^7^ cells/well were seeded into fibronectin-coated six-well plates and cultured in endothelial cell growth medium-2 (EGM-2) with supplements (Lonza, Milan, Italy) at 37 °C in a humidified 5% CO_2_ incubator for 7 days. On day 7, adherent cells displaying a spindle-shaped morphology were considered MACs.

In order to assess a potential beneficial effect of bergamot-derived flavanone metabolites on lipotoxicity, MACs were incubated with physiological concentrations (100 µM) of SA according to our previous study [11]. Stearate stock solution was prepared by dissolving SA in 0.1 M NaOH at 72 °C for 30 min, then 5 mM stearate was complexed to 10% bovine serum albumin (BSA) (FFA:BSA molar ratio = 3.3:1) as reported in literature [32].

SA-treated MACs were incubated in the presence/absence of those molecules identified in the bioavailability study (naringenin-7-*O*-glucuronide (N7G), naringenin-4′-*O*-glucuronide (N4G), hesperetin-7-*O*-glucuronide (H7G) and hesperetin-3′-*O*-glucuronide (H3G)) at the physiological concentration of 1 µM [33], in line with data recorded herein. Vehicle (DMSO 0.1%) was used as control.

### 2.7. Pro-Inflammatory Marker Gene Expression

Quantitative PCR (qPCR) assays were used to assess pro-inflammatory marker gene expression in MACs pre-incubated with N4G, N7G, H3G and H7G for 16 h and then exposed to SA for 3 h. Cells were lysed with QIAzol lysis reagent and total RNA was extracted using miRNeasy Mini Kit (both from Qiagen Ltd., West Sussex, UK) and quantified by NanoDrop (NanoDrop Technologies, Wilmington, DE, USA). iScript Reverse Transcription Kit (Bio-Rad Laboratories, Inc., Hercules, CA, USA) was used to obtain cDNA, starting from 250 ng of total RNA. Interleukin (IL)-1β, IL-6, IL-8 and tumor necrosis factor-α (TNF-α) gene expression was assessed using SsoAdvanced Universal Probes Supermix (Bio-Rad) with TaqMan primers and probes (Applied Biosystems, Carlsbad, CA, USA) on a CFX Connect Real-Time (Bio-Rad), as already reported [30]. Specific thermal cycling conditions were used: 98 °C for 30 s, followed by 40 amplification cycles (95 °C for 3 s; 60 °C for 20 s). Gene expression values were calculated based on the ΔΔCt method using GAPDH as reference gene. Eight independent experiments were performed and samples were analyzed in triplicate.

### 2.8. Apoptosis Assessment

The potential effects of the tested metabolites on SA-induced apoptosis were assessed by Caspase-Glo 3/7 assay, according to manufacturer’s instructions (Promega Corporation, Madison, WI, USA). MACs were cultured in 96-well culture plates (2.5 × 10^5^ cells/well) and pre-incubated with the tested metabolites 16 h before being exposed to stearate for 24 h. Cells were then incubated with 100 μL of Caspase-Glo 3/7 reagent at 37 °C for 30 min and luminescence was measured by Cary Eclipse fluorescence spectrophotometer (Varian/Agilent, Santa Clara, CA, USA). Fold increase in caspase activity was normalized to the activity obtained from stearate-treated cells.

### 2.9. Statistical Analysis

All data are presented as mean ± SEM (standard error of the mean), except for the phenolic composition of the BJ, expressed as mean ± SD (standard deviation). Differences between groups were identified using one-way ANOVA followed by Dunnet’s post-hoc test (with SA as reference condition). Statistical significance was set at *p* < 0.05 (two-sided). Data analysis was performed using SPSS version 24.0 (SPSS Inc./IBM, Chicago, IL, USA).

## 3. Results

### 3.1. Flavanone Composition of the Bergamot Juice

A total of 16 flavanones were identified and quantified in the BJ (Table 1). Five compounds corresponded to naringenin derivatives (compounds **3**, **5**, **8**, **9** and **13**), four different derivatives were found for both eriodyctiol (**1**, **2**, **6** and **16**) and hesperitin (**4**, **7**, **10** and **15**) and three isosakuranetin compounds (**11**, **12** and **14**) were identified. Most of the compounds detected were rutinosides or neohesperidosides of the aforementioned aglycones, while four compounds were 3-hydroxy-3-methylglutaryl (HMG) glycosides. Three compounds were identified by comparison with their commercial reference standards, while the remaining 13 flavanones were tentatively identified by interpreting and comparing their mass spectra, obtained from MS^2^ and MS^3^ experiments, with data from the literature [34,35,36,37]. A new HMG flavanone (compound **12**) was tentatively identified for the first time, to the best of our knowledge, in bergamot juice. This flavanone, isosakuranetin-7-*O*-neohesperidoside-6″-*O*-HMG (*m*/*z* 737), showed the characteristic fragmentation pattern of HMG derivatives according to Salerno et al. [35]: fragment ions at *m*/*z* [M − H − 62]^−^, [M − H − 102]^−^ and [M − H − 144]^−^. It also exhibited a fragment ion at *m*/*z* 285, which was identified as isosakuranetin by a further MS^4^ experiment attending to its characteristic fragmentation spectra. Some flavones previously reported in BJ [34,35] were also identified in the BJ (data not shown).

The main flavanones in BJ were brutieridin, naringin, neohesperidin, melitidin, neoeriocitrin and eriodictyol-7-*O*-neohesperidoside-6″-*O*-HMG (Table 1). Small quantities of the other glycosides identified were also detected. With regard to the amount of specific flavanone aglycones, hesperetin derivatives accounted for the 44% of the flavanone composition, naringenin derivatives comprised the 30% and eriodyctiol derivatives the 24%, while isosakuranetin derivatives made up the remaining 2%.

### 3.2. Flavanone Metabolites in Plasma and Urine Samples

Up to 12 structurally related flavanone metabolites were identified in plasma and urine samples after BJ consumption. Their UHPLC retention times and mass spectra, as well as their occurrence in plasma and urine, are presented in Table 2. Some of these metabolites were identified by comparison with commercially available reference compounds, while the criteria for the identification of the rest of the compounds were based on previous studies [39,40,41]. Glucuronide and sulfate phase II metabolites were identified through the loss of the conjugation moiety (*m*/*z* 176 and 80 for glucuronides and sulfates, respectively). Successive MS fragmentation of the aglycone was performed to confirm the identification of the metabolites through their characteristic fragmentation patterns [38]. Five metabolites were hesperetin conjugates (compounds **M1**, **M2**, **M7**, **M8** and **M12**), 4 naringenin conjugates (**M4**, **M5**, **M9** and **M11**) and 3 eriodictyol derivatives (**M3**, **M6** and **M10**). No isosakuranetin conjugates were found.

Most of the flavanone phase II conjugates were recorded in both biological fluids. They were not recorded at baseline, while they were detected at both 1 h and 4 h in the plasma samples of all the volunteers. The majority of them were also detected in urine at 2 h and 6 h, accounting for the absorption profile observed in plasma. The main flavanone metabolites in plasma were sulfate conjugates of hesperetin and eriodyctiol, followed by glucuronides of hesperetin and naringenin (Table 3). Plasma concentrations for these compounds ranged from 0.11 to 3.54 μM, while the rest of the compounds presented concentrations below 0.1 μM. Despite the reduced number of subjects considered, a certain degree of inter-individual variability was observed, being notably higher at 4 h than at 1 h (mean variability of 23% and 69% at 1 h and 4 h, respectively).

### 3.3. Effects of Selected Flavanone Metabolites on the Gene Expression of Inflammation Markers

**Figure 1:**
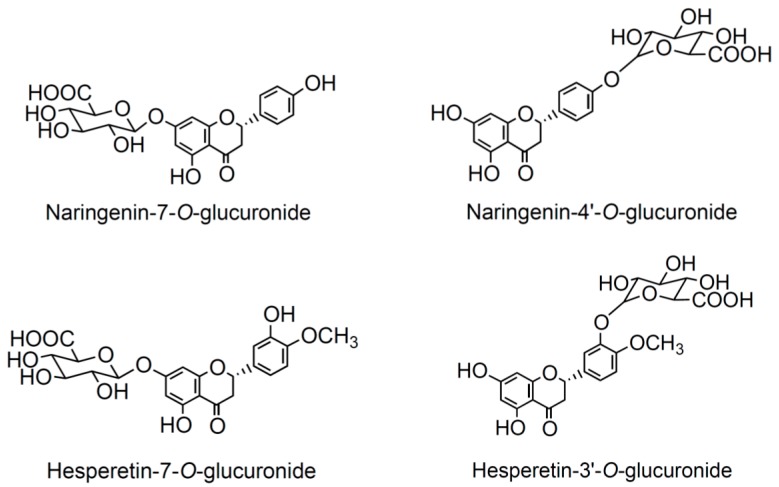
Molecular structures of tested compounds.

On the basis of the metabolites found in plasma of healthy volunteers and considering the limited commercial availability of phase II conjugates of flavanones, four glucuronides (two isomers of hesperetin and two of naringenin) were tested on MACs exposed to SA (Figure 1). The concentrations were selected taking into account the concentrations found in the study in humans and previous literature reporting on the concentration of these metabolites after consumption of other citrus fruit juices [33].

**Figure 2:**
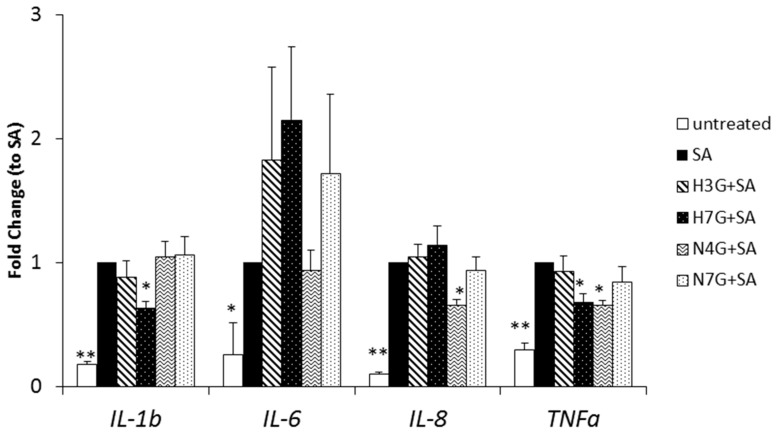
Flavanone metabolites reduce stearate-induced inflammation in MACs. Pro-inflammatory marker (IL-1β, IL-6, IL-8 and TNF-α) gene expression in MACs pre-incubated with 1 µM of naringenin-7-*O*-glucuronide (N7G), naringenin-4′-*O*-glucuronide (N4G), hesperetin-7-*O*-glucuronide (H7G) and hesperetin-3′-*O*-glucuronide (H3G) for 16 h and then exposed to stearate (SA, 100 µM) for 3 h is shown. Vehicle (DMSO 0.1%) was used as control (untreated). Real time PCR data are normalized to *GAPDH* housekeeping gene and expressed as fold induction of SA culture condition. The results represent means ± SEM (* *p* < 0.05 vs. stearate; ** *p* < 0.01 vs. stearate; *n* = 8).

Gene expression of pro-inflammatory markers was assessed to evaluate whether H3G, H7G, N4G and N7G were effective in blunting lipotoxic damage in MACs. As expected, gene expression of IL-1β, IL-6, IL-8 and TNF-α increased following stearate exposure (100 µM, 3 h) (Figure 2). Of note, H7G curbed IL-1β (by 37 ± 6%; *p* < 0.05) and TNF-α (−32 ± 7%; *p* < 0.05) gene expression. Exposure to N4G mitigated mRNA expression of IL-8 and TNF-α (both by 34 ± 4%; *p* < 0.05) in MACs following SA exposure. Neither H3G nor N7G had any significant effect on the gene expression of the aforementioned pro-inflammatory markers.

### 3.4. Effects of Flavanone Metabolites on Cell Apoptosis

Pilot experiments confirmed that none of the tested molecules induced cytotoxic effects in MACs at 1 µM concentration (data not shown). The effect of H3G, H7G, N4G and N7G in curbing caspase 3 and 7 activation in SA-treated MACs was evaluated in order to detect whether bergamot-derived phenol metabolites may also have anti-lipoapoptotic properties.

**Figure 3:**
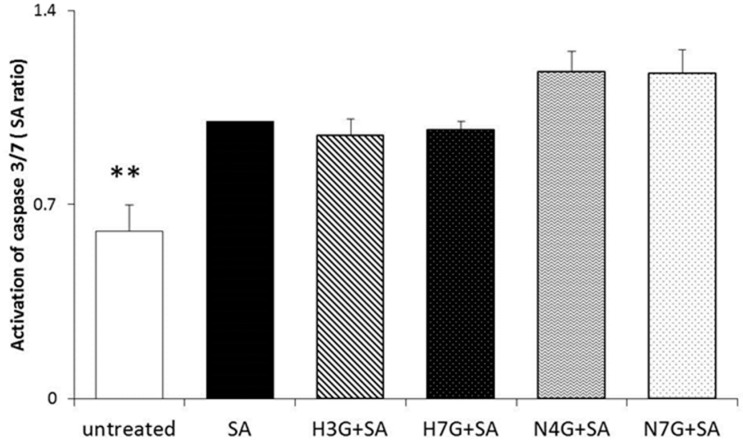
Effects of flavanone metabolites on apoptosis. Caspase 3/7 activation in myeloid angiogenic cells (MACs) pre-incubated with naringenin-7-*O*-glucuronide (N7G), naringenin-4′-*O*-glucuronide (N4G), hesperetin-7-*O*-glucuronide (H7G) and hesperetin-3′-*O*-glucuronide (H3G) at 1 µM for 16 h and then exposed to stearate (SA, 100 µM) for 24 h. Vehicle (DMSO 0.1%) was used as control (untreated) and results are expressed as means ± SEM (** *p* < 0.01 vs. stearate; *n* = 8).

No effects in reducing stearate-induced activation of caspases 3 and 7, compared to vehicle treated cells, was shown following metabolite addition in culture at the physiological concentration tested (Figure 3).

## 4. Discussion

This study presents a comprehensive approach focused on exploring the bioactivity of the metabolites in circulation after consumption of sources of phenolic compounds. As required to fully pinpoint the responsible bioactive(s) and mechanisms of action in the extremely complex physiological scenario, the study was conducted by applying integrated multidisciplinary lenses: first, a detailed characterization of the food matrix; second, the study in humans of the characteristic transformations of the compounds found in food; and third, the study of the biological activity of the compounds in circulation, tested at physiologically achievable concentrations.

The characterization of the (poly)phenolic fingerprint of BJ had been addressed in recent years [34,35]. It is composed mainly of flavanones and relevant amounts of flavones. However, among all its compounds, it was mainly the specific composition of bergamot in 3-hydroxy-3-methylglutaryl-glycoside flavonoids to attract the attention of the research community during the last decade [20,36,42]. Interestingly, despite the accurate works carried out in the past, it was possible to identify, for the first time, a new HMG flavanone, isosakuranetin-7-*O*-neohesperidoside-6″-*O*-HMG (compound **12**). Although additional analytical techniques are required to fully confirm the structure of this molecule (i.e., NMR), we proposed it to be named “parmigin.” This is a point worth mentioning since it extends the knowledge on the phenolic composition of bergamot and citrus plant biology. On the contrary, a compound previously reported in bergamot products at high concentrations, isosakuranetin-7-*O*-neohesperidoside (poncirin) [34,43], was not detected in the present work; although the flavone luteolin-rutinoside/neohesperidoside, which shares a similar fragmentation pattern with poncirin and has been previously reported in bergamot [35], was detected. This could be due to differences in the methodological identification of this compound or to some factors affecting the phenolic composition of citrus products like juice processing or pre-harvest conditions [44].

The bioavailability study accounted for the extensive presence of phase II conjugated flavanone metabolites 1 and 4 h after consumption of BJ. The occurrence of these metabolites in circulation is related to the cleavage of the glycoside units of the compounds present in the juice at intestinal level and the subsequent conjugation of the flavanone aglycones with glucuronide and sulfate moieties by phase II enzymes [39,41,45]. So far, no studies on the bioavailability of BJ flavanones had been conducted. This study has allowed to describe the absorption profile of BJ flavanones during the first few hours after BJ consumption. The profile of flavanone metabolites in plasma matched the pharmacokinetic profiles previously reported for these compounds [39,41,44,45,46,47]. With respect to the absorption and metabolism of the characteristic HMG-containing flavanones of bergamot, they were not found in plasma or urine samples despite they were selectively targeted. The lack in circulation of these compounds may indicate that they undergo the same transformations of glycosylated flavanones, circulating thus as phase II conjugates. Nevertheless, their appearance at time points beyond 4–6 h cannot be ruled out, although it does not seem plausible considering the metabolic steps undergone by flavanones with different glycoside moieties [45,48,49]. Last, the inter-individual variability observed in the plasmatic concentrations of flavanone conjugates is in line with a recent study [50]. Nevertheless, data on inter-individual variability should be intended to be of preliminary nature, considering the low number of subjects involved in this study, enough to identify the metabolites and concentrations to be tested but not to properly address this topic.

Substantial clinical evidence supports the beneficial vascular effects of flavanones. Randomized-controlled studies showed that hesperidin (500 mg/day for 3 weeks) [51] and flavanone-rich citrus (orange) beverage [52,53] intake was associated with an improvement in endothelial function in healthy subjects [52,53] and in individuals with metabolic syndrome [51]. Of note, in one of these studies [52], the improvement in flow mediated dilation coincided with the peak of naringenin/hesperetin metabolites in circulation, supporting a causal relationship. Some results have also been published reporting the effects of BJ and its flavanones on the CV system [20,34,42,54]. BJ has been reported to exert anti-inflammatory activities [21], as recently demonstrated in an experimental model of bowel disease via modulation of several pathways [55]. 

In mechanistic studies, BJ reduced LPS-induced pro-inflammatory response (IL-1β, IL-6 and TNF-α) in THP-1 macrophages by decreasing NF-kB activation [23]. The same authors reported a similar anti-inflammatory effect on THP-1 exposed to amyloid-beta, associated with up-regulation of ERK1/2 and JNK MAPK [22]. However, the above cited works are fraught with methodological concerns as cultured cells were either exposed to flavonoid levels far from those achievable in the bloodstream, or to BJ itself, which obviously represents a non-physiological condition with evident misleading and paradoxical results. A methodological strength of the present study is that the cell experiments were carried out by treating cells with the metabolites identified in circulation following BJ intake in humans. Moreover, the concentrations tested, although slightly higher than those registered at 4 h, are in line with concentrations achievable after citrus juice consumption or that could even be achievable at longer time points [33,45].

To date, there is a paucity of studies investigating the effects of phase II flavonoid metabolites at physiological concentrations on the mechanisms underlying their possible benefits on vascular biology. Physiological concentrations of naringenin and hesperetin glucuronides have been reported to reduce migration and inflammation in human aortic endothelial cells [56], monocyte adhesion to umbilical vein endothelial cells activated by TNF-α [57] and gene expression in differently activated human macrophages [58]. This study expands these findings demonstrating that these metabolites also reduce pro-inflammatory cytokine expression (i.e., IL-1β, IL-8 and TNF-α) in key primary cells mediating vascular reparative processes. In particular, our novel findings show that some BJ flavanone circulating derivatives (naringenin-4′-*O*-glucuronide and hesperetin-7-*O*-glucuronide) improve MAC function in terms of inflammatory cytokine activation under lipotoxic conditions. However, these compounds were not effective in curbing stearate-induced lipoapoptosis at the tested concentrations. A potential anti-apoptotic/pro-survival effect of these compounds at higher metabolite levels or in a concomitant presence cannot be excluded. In general, these results are particularly significant when considering (a) the variety of conditions and diseases associated to increased SA levels and thus lipotoxicity, such as IR, obesity and diabetes [5]; (b) the growing evidence of the role of dietary compounds, including citrus polyphenols, in the prevention/treatment of metabolic and (cardio)vascular diseases [14,59]; and (c) the pivotal role played by MACs in the maintenance of vascular integrity, emphasized by their role in the prediction of CV events and mortality in humans [3]. Nevertheless, despite the interest of these novel findings, whether bergamot consumption can display anti-atherogenic actions by improving endothelial regenerative capacity when challenged by a lipotoxic clue is a question that should be answered with further in vivo studies. In the light of these results, not only bergamot but also other citrus fruits might exert this bioactivity.

The observation that only N4G and H7G (and not N7G and H3G) modulated SA-induced inflammation suggests that the biological action of naringenin and hesperetin glucuronide metabolites might depend on the position of the conjugation, as already hypothesized [56,60]. Our results are in accordance with the work by Yamamoto and colleagues in which H7G but not H3G, decreased H_2_O_2_-induced pro-inflammatory markers (intracellular adhesion molecule-1 and monocyte chemoattractant protein-1) gene expression in endothelial cells [60]. The different activity on the expression of selected genes in human macrophages of naringenin metabolites depending on the glucuronide position had also been previously reported [58].

In conclusion, these results demonstrated that some flavanone metabolites, derived from the metabolism of BJ phenolics in humans, ameliorated SA-induced inflammation in MACs. To better understand the prospects of bergamot cardiovascular protection, further intervention studies are required. Future bioavailability studies should assess longer periods of time (up to 24/48 h), evaluate the production of colonic metabolites and take into account inter-individual differences among volunteers. Randomized controlled studies with bergamot juice/extract on recognized markers of CV health are also needed. Lastly, the underlying molecular mechanism behind the bioactivity observed remains uninvestigated and future studies on this regard should be performed.

## Figures and Tables

**Table 1:** Flavanone compounds in bergamot juice.

Id.	Compounds	RT (min)	[M − H]^−^ (*m*/*z*)	MS^2^ Ion Fragments (*m*/*z*) ^a^	MS^3^ Ion Fragments (*m*/*z*) ^a^	Ident. ^c^	Concentration (μmol/g dw)
**1**	Eriodictyol-7-*O*-rutinoside (eriocitrin)	9.5	**595**	**287** ^b^	151	[34]	2.30 ± 0.22
**2**	Eriodictyol-7-*O*-neohesperidoside (neoeriocitrin)	9.71	**595**	**459**, 287, 235	235, 357, 441, 271, 151	[34]	7.83 ± 0.85
**3**	Naringenin-7-*O*-rutinoside (narirutin)	10.19	**579**	**271**	151, 177	Std	1.06 ± 0.05
**4**	Hesperetin-7-*O*-rutinoside (hesperidin)	10.2	**609**	**301**	286, 242, 283, 257, 125	Std	2.10 ± 0.22
**5**	Naringenin-7-*O*-neohesperidoside (naringin)	10.4	**579**	**459**, 271, 235	357, 235, 271, 441, 339	[38]	12.46 ± 1.61
**6**	Eriodictyol-7-*O*-neohesperidoside-6″-*O*-HMG	10.57	**739**	**595**, 637, 677	459, 287	[35]	7.53 ± 0.76
**7**	Hesperetin-7-*O*-neohesperidoside (neohesperidin)	10.79	**609**	**301**, 343, 489, 447	286, 242, 283, 257, 125	[34]	10.31 ± 0.61
**8**	Naringenin-7-*O*-neohesperidoside-6″-*O*-HMG (melitidin)	11.13	**723**	**579**, 621, 661	459, 271, 313	[34]	8.25 ± 0.93
**9**	Naringenin-7-*O*-glucoside-6″-*O*-HMG	11.39	**577**	**271**, 433, 475	151, 177	[35]	0.27 ± 0.03
**10**	Hesperetin-7-*O*-neohesperidoside-6″-*O*-HMG (brutieridin)	11.43	**753**	**609**, 651, 691	301, 343, 489	[34]	19.70 ± 2.04
**11**	Isosakuranetin-7-*O*-rutinoside (didymin)	12.25	**593**	**285**, 327, 473	270, 243, 164, 241, 151	Std	0.26 ± 0.02
**12**	Isosakuranetin-7-*O*-neohesperidoside-6″-*O*-HMG (parmigin)	12.75	**737**	**593**, 635, 675	**285** (MS^4^: 270, 243), 327, 473	-	0.99 ± 0.03
**13**	Naringenin	13.32	**271**	151, 177		Std	traces
**14**	Isosakuranetin	13.57	**285**	270, 243		Std	traces
**15**	Hesperetin	13.78	**301**	286, 242		Std	traces
**16**	Eriodyctiol	13.82	**287**	151		[38]	traces

^a^ Fragment ions are listed in order of relative abundance; ^b^ MS ions in bold were those subjected to successive MS fragmentation; ^c^ Ident., identification mode: [Reference] or Std (standard, compound identified by comparison of its retention time and MS data with that of a reference compound). Mean (*n* = 3) ± SD. RT, retention time.

**Table 2:** Flavanone metabolites in plasma and urine collected after consumption of bergamot juice (BJ).

Id.	Compounds	RT (min)	[M − H]^−^ (*m/z*)	MS^2^ Ion Fragments (*m/z*) ^a^	MS^3^ Ion Fragments (*m/z*) ^a^	MS^4^ Ion Fragments (*m/z*) ^a^	Location ^c^
**M1**	Hesperitin-*O*-glucuronide-sulfate	3.32	**557**	**381** ^b^, 447	**301**	286, 242, 199, 283	P
**M2**	Hesperitin-*O*-glucuronide-sulfate	3.51	**557**	**381**, 447	**301**, 229	286, 242, 199, 283	P, U
**M3**	Eriodictyol-*O*-glucuronide	4.04	**463**	**287**, 175	151		P, U
**M4**	Naringenin-7-*O*-glucuronide	4.09	**447**	**271**, 175	151, 177		P, U
**M5**	Naringenin-4′-*O*-glucuronide	4.23	**447**	**271**, 175	151, 177		P, U
**M6**	Eriodictyol-*O*-glucuronide	4.23	**463**	**287**, 175	151, 269		P, U
**M7**	Hesperitin-7-*O*-glucuronide	4.45	**477**	**301**, 175	286, 243, 283		P, U
**M8**	Hesperitin-3′-*O*-glucuronide	4.63	**477**	**301**, 175	286, 243, 283		P, U
**M9**	Naringenin-sulfate	4.68	**351**	**271**	151, 177		P
**M10**	Eriodictyol-sulfate	4.78	**367**	**287**	151		P, U
**M11**	Naringenin-sulfate	4.82	**351**	**271**	151, 177, 165		P
**M12**	Hesperetin-sulfate	4.9	**381**	**301**	286, 243, 283, 199		P, U

^a^ Fragment ions are listed in order of relative abundance; ^b^ MS ions in bold were those subjected to successive MS fragmentation; ^c^ P, plasma; U, urine. RT, retention time.

**Table 3:** Concentration of flavanone metabolites in plasma after consumption of bergamot juice ^a^.

Id.	Compounds	1 h		4 h	
		Mean ± SEM	CV	Mean ± SEM	CV
**M1**	Hesperitin-*O*-glucuronide-sulfate	0.021 ± 0.004	19%	0.019 ± 0.006	31%
**M2**	Hesperitin-*O*-glucuronide-sulfate	0.033 ± 0.008	24%	0.094 ± 0.093	98%
**M3**	Eriodictyol-*O*-glucuronide	0.096 ± 0.010	10%	0.085 ± 0.022	26%
**M4**	Naringenin-7-*O*-glucuronide	0.169 ± 0.032	19%	0.163 ± 0.042	26%
**M5**	Naringenin-4′-*O*-glucuronide	0.183 ± 0.017	9%	0.194 ± 0.050	26%
**M6**	Eriodictyol-*O*-glucuronide	0.095 ± 0.016	16%	0.087 ± 0.045	52%
**M7**	Hesperitin-7-*O*-glucuronide	0.112 ± 0.011	10%	0.162 ± 0.091	56%
**M8**	Hesperitin-3′-*O*-glucuronide	0.177 ± 0.020	11%	0.411 ± 0.355	86%
**M9**	Naringenin-sulfate	0.085 ± 0.050	59%	0.100 ± 0.091	91%
**M10**	Eriodictyol-sulfate	0.620 ± 0.195	31%	0.960 ± 0.935	97%
**M11**	Naringenin-sulfate	0.059 ± 0.016	28%	0.058 ± 0.059	102%
**M12**	Hesperetin-sulfate	0.451 ± 0.163	36%	3.542 ± 4.562	129%

^a^ Data expressed in μM (*n* = 3). CV, coefficient of variation.
